# Optimal search strategies on complex multi-linked networks

**DOI:** 10.1038/srep09869

**Published:** 2015-05-07

**Authors:** Francesca Di Patti, Duccio Fanelli, Francesco Piazza

**Affiliations:** 1 Università degli Studi di Firenze, Dipartimento di Fisica e Astronomia and CSDC, via G. Sansone 1, 50019 Sesto Fiorentino, Firenze, Italia; 2 INFN, Sezione di Firenze, Italia; 3 Université d'Orléans, Centre de Biophysique Moléculaire, CNRS-UPR4301, Rue C. Sadron, 45071, Orléans, France

## Abstract

In this paper we consider the problem of optimal search strategies on multi-linked networks, i.e. graphs whose nodes are endowed with several independent sets of links. We focus preliminarily on agents randomly hopping along the links of a graph, with the additional possibility of performing non-local hops to randomly chosen nodes with a given probability. We show that an optimal combination of the two jump rules exists that maximises the efficiency of target search, the optimum reflecting the topology of the network. We then generalize our results to multi-linked networks with an arbitrary number of mutually interfering link sets.

Complex networks are ubiquitous in nature and play a role of paramount importance in many contexts. Internet and the cyberworld, which permeate our everyday life, are self-organized hierarchical graphs. Urban traffic flows on intricate road networks, which impact both transportation design and epidemic control. In the brain, neurons are cabled through heterogeneous connections, which support the propagation of electric signals. In all these cases, the true challenge is to unveil the mechanisms through which specific dynamical features are modulated by the underlying topology of the network. Here, we study the utterly general problem of optimal search strategies on multi- linked networks, i.e. graphs which have independent sets of edges that connect the same group of nodes.

To this aim, let us start by considering a given agent (*e.g.* an electric pulse, an excitation, an animal or a human individual, such as a web surfer) located at a node of a network. The agent can hop to a neighbouring node, provided a link exists as specified by the adjacency matrix associated with the graph. The walker wanders on the network through a sequences of steps, that allow for a *local* exploration of its support. In such situations, the efficiency in reaching a specified location may be quantified by the mean first passage time, a robust and widely used measure of transport efficiency on networks in many contexts^5^,^4^, from biology^19^ and ecology^8^,^10^,^40^ to road network dynamics^17^ and quantum systems^38^,^16^.

However, local moves are not always the best option to reach a target efficiently. For example, facilitated diffusion in the cell nucleus, a mix of one-dimensional gliding along the DNA and three-dimensional jumps to adjacent DNA strands, is believed to account for the efficiency of transcription factors in finding their binding sites^7^,^32^,^9^. Analogously, inspired by the behaviour of foraging animals, it has been hypothesised that the *local* exploration of a connected territory might be complemented by intermittent relocation phases in order to optimize the searching strategy^23^. Accordingly, the animal would venture off-track through ballistic runs from time to time, thus sampling larger portions of space. In such examples, the relative duration of the local and relocation stages may control the optimization of the dual-stage strategy^36^.

Walkers on complex networks could in principle rely on similar integrated strategies, possibly tuned to the heterogeneous nature of the underlying support. In the seminal paper by Kleinberg^25^, the navigation on a regular two dimensional lattice distorted with the inclusion of long-range connections was studied and an optimum for the delivery time found, when tuning the probability of off-lattice connections between nodes. Exacts results and new insights on the navigability of small-world networks were later reported independently in Refs. 14 and 15. Since the pioneering contribution by Kleinberg, the problem of assisted exploration of a network has been addressed in a number of different contexts^22^,^26^,^28^,^29^,^30^,^35^,^37^, mainly relying on numerical investigations targeted to the problem at hand.

To help intuition let us consider, for example, web surfing. Starting the exploration from an arbitrary web page, one usually proceeds by following the hyperlinks which are therein available. This is a *local* search, which the user abandons when she opens a new tab to look for a different, potentially related topic, eventually landing into another virtual compartment which will be again probed locally for some time. On a different level, the brain displays multi-layered architectures of connections, that assist the finely orchestrated spatio-temporal patterns underlying brain function^6^. One may then speculate that electric signals can be transmitted across different layers, thus realizing *de facto* non-local jumps in the overall brain connectome between single-layer connected components. Non-local jumps (moves directed towards target sites which are not physically linked to the departure node) may be also loosely referred to as *long-range*, even though the underlying network is not necessarily endowed with a metric.

Building upon these ideas, we start by investigating the conditions for optimal target searches on a generic network of 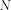 nodes. In order to quantify search efficiency on a given network, we shall analytically compute mean first passage times^31^,^34^,^24^, which are widely used to gauge search strategies in many contexts^42^,^2^,^21^,^46^. To investigate the combined effect of local and non-local moves, we study a simple stochastic process which accommodates for both local diffusion and non-local relocation to sites which are not connected through the adjacency matrix.

An interesting application of such analysis is random walk on two networks that share the same nodes but are endowed with two different sets of links. Altogether, the two nested graphs sharing the same nodes might be termed a *multi-linked* network. Only local moves are allowed in this case, as prescribed by the two adjacency matrices associated with the two link sets. Moving from this observation, in the second part of the paper we will examine a more general setting where agents can diffuse in multi-linked networks endowed with an arbitrary number of independent link sets. The ensuing sub-networks can be imagined to be assembled as a stratified hierarchy of independent levels, each hosting an identical replica of the nodes, as in the spirit of a multiplex^20^,^11^,^33^,^1^. Indeed, it is straightforward to extend our analysis to the relevant, and more general situation where only a subset of the nodes is present in each nested level.

## Results

### Relocation-assisted diffusion

Let 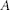 denote the 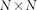 adjacency matrix of the network, with 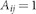 if 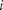 and 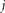 are physically connected by a link, and 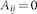 otherwise. The degree of node 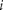 is given by 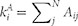. The probability that a particle sitting at node 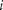 jumps on any other node 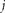 is specified by the following matrix
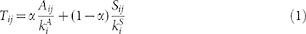
where 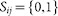 are the entries of a random symmetric sparse 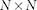 matrix, that controls the relocation via non-local hops (self-loops are, in principle, also allowed for). The density of ones in 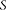 is measured by the parameter 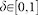, so that the average number of nodes that can be reached from any node 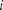 via off-network long-range jumps is 
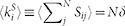
. The parameter 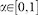 tunes the relative strength of the two competing mechanisms, local diffusion and random relocation. When 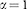 the walker explores the network according to a purely local rule, while in the opposite limit, 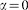, hopping towards disconnected sites are the only allowed moves. For 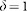, the matrix 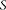 is filled with ones and 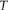 becomes the known Google matrix used in the PageRank Algorithm^12^,^27^.

We define the search time as the time needed by a particle starting at node 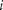 to reach an absorbing trap located at node 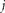. This satisfies the following relation (see Methods)
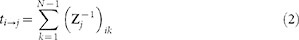
where 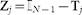. The subscript 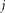 indicates an 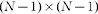 submatrix obtained by suppressing the 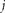-th row and the 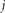-th column, while 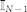 denotes the identity matrix of size 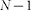. To assess the overall ability of the walker to find a target, we introduce a global parameter 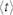 by averaging Eq. (2) over all possible starting nodes (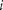) and trap locations (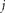), that is,
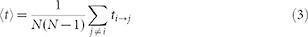


In short, 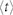 quantifies the ability of the walker to search for targets at the global scale of the network. The shorter 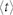, the more efficient the search. The quantity 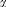 acts as a free parameter – it can be adjusted to select the optimal balance between local and non-local hops, with the aim of minimizing the global exploration time.

**Figure 1:**
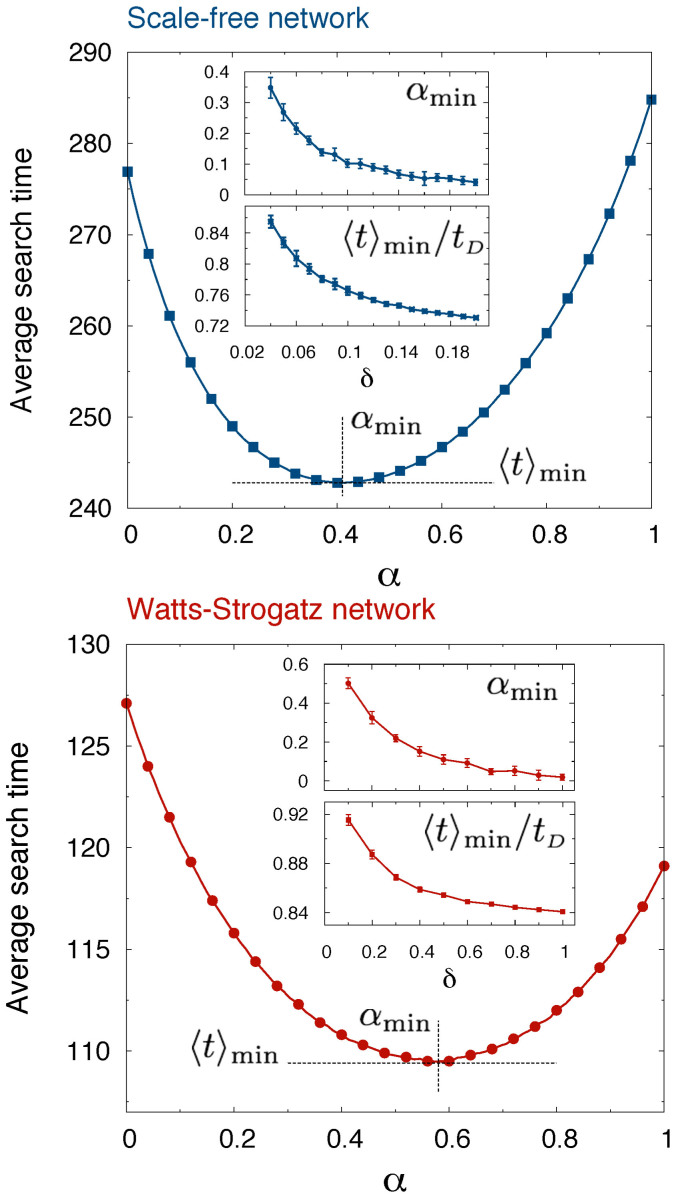
The average search time on synthetic networks displays an optimum as a function of the relative weight of local and non-local moves. Upper panel: scale-free network generated with the preferential attachment method^3^ with 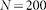 and average connectivity 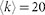. Lower panel: Watts and Strogatz small-world random network with 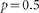
44, 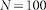 and average connectivity 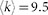. Here the sparse symmetric matrix 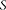 has been generated with 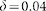 (scale-free) and 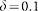 (Watts-Strogatz). The insets show the position of the minimum 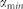 and the corresponding shortest average time 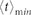 (normalized to the case of a purely local walker, 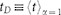) as a function of the average fraction of long-range accessible nodes 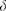. The data are averaged over 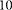 independent realizations of the random matrices 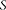 and error bars correspond to one standard deviation.

Figure 1 illustrates how 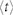 changes as a function of the relative weight of local and non-local moves for two different classes of synthetic undirected networks, the scale-free^3^,^13^ and the small-world^44^ networks. The curves display a clear minimum, implying the existence of an optimal value of 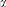 which minimizes the search time. Exactly the same behaviour is displayed by directed networks. The location of the minimum depends on the topology of the network, which defines the backbone for local diffusion, but also on the average number of sites that can be reached through a single long-range hop, 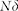. Remarkably, the fewer sites are accessible through non-local jumps (*i.e.* the smaller 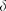), the more pronounced the optimality condition (see upper insets in Fig. 1).

When 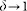, 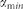 approaches (but never reaches) the limiting solution 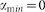. In this case, the walker can virtually land on any node with just one jump (the matrix 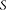 is completely filled with ones), and local diffusion contributes modestly to further reduce the average searching time. Although a minimum always exists also for 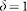 (the Google Matrix case), 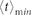 is very close to 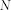, the time the walker needs to reach an isolated trap when 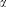 is exactly set to zero. Conversely, when 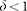, non-local short-cuts are only available towards a subset of nodes. This is a more plausible situation, bearing in mind the afore-mentioned applications. When surfing the web, from time to time one will abandon a given area of exploration to look for the presumed central node of a new region that she wishes to sample. Similarly, long-range connections in the brain, established through trans-layer channels, are certainly fewer than those accounting for effective bridges among the 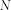 nodes of a given layer.

The relocation-assisted search is 10–15% more efficient with respect to the purely local dynamics for intermediate values of the density 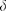 of available distant nodes (insets in Fig. 1). The same analysis performed with different values of the average connectivity 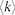 (scale-free network) and of the rewiring parameter *p* (Watts-Strogatz) yields similar results. In particular, upon decreasing 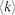 one recovers the same qualitative behaviour as obtained when increasing 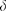 (data not shown). In this paper, we have chosen to focus on small networks because of the computational cost required to invert the matrix **Z***_j_*, for any given *j*. However, the method is utterly general and may be applied to systems of arbitrary size *N*. In the Supplementary Information, we show that 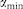 progressively decreases as *N* increases, while keeping 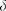 constant. Conversely, working at fixed size *N* one can change the position of the minimum as sought, by acting on 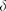. The counter-intuitive conclusion is that the density of non-local links should be reduced as *N* gets larger to keep the optimum value of 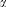 fixed. Interestingly, the advantage brought about by the assisted search over purely local diffusion is preserved if *N* and 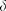 are simultaneously tuned so to keep 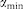 fixed. The data reported in the Supplementary Information refer to the Watts-Strogatz setting but the same conclusions hold in general. As a side remark, we recall that the searching time can be expressed as a function of the nodes connectivity, for specific classes of networks, in the large *N* limit^43^. Such large networks could share the same characteristics of searchability for the limiting choice 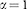. When introducing the effect of long-range teleportation one can in principle remove such degeneracy and obtain distinct optimal values of 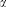.

**Figure 2:**
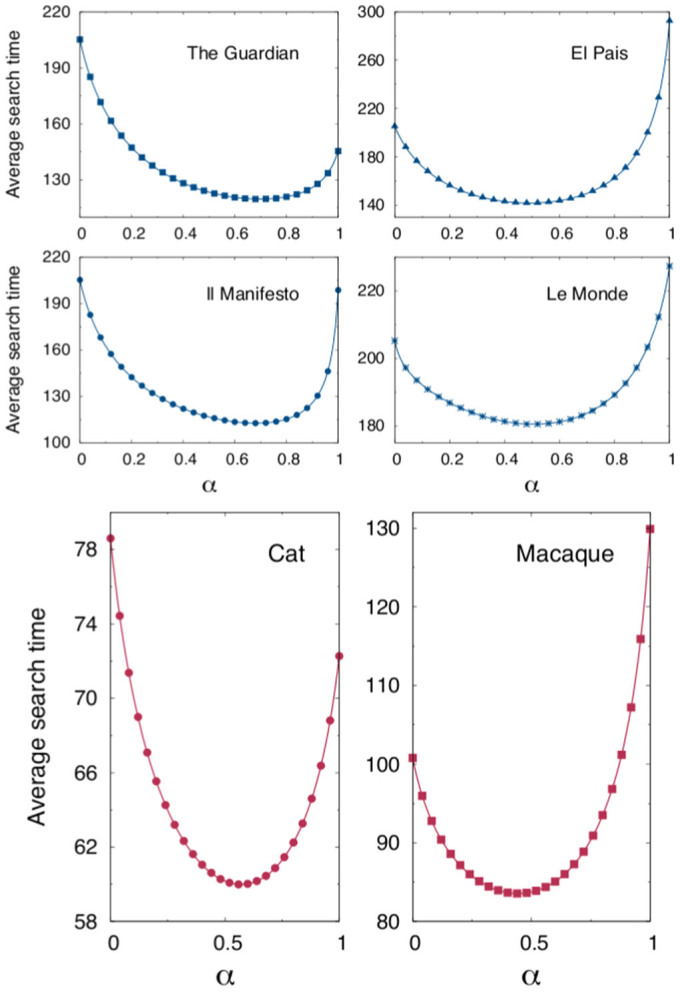
The average search time on real data sets displays an optimum as a function of the relative weight of local and non-local moves. Top: average time 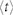 as a function of 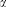 for four real Web subgraphs. The 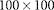 adjacency matrices have been mapped out by a Web crawler starting from the web sites of four major European newspapers (see Methods). The sparse symmetric matrix 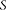 has been generated with 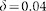. Bottom: search time in two neuronal networks: cortical connectivity network of cats (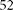 nodes, left^39^) and macaques (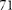 nodes, right^45^). In both cases we have used 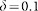.

To confirm the existence of an optimal searching strategy on real data sets, we have extracted the adjacency graph of small portions of the web, starting from the home-pages of four main European newspapers (see Methods). The top panel of Fig. 2 shows that the general picture described above for synthetic data sets is valid for real networks too. This has nothing to do with the peculiar structure of the Web, for the same analysis performed on small neural networks of different animals confirms the existence of a clear minimum in the average search time (bottom panel in Fig. 2).

In all the cases examined, 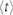 appears to be a convex smooth function of 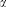 with a clear minimum. One may ask whether this is a widespread feature of many graphs. More generally, it would be helpful to work out a quantitative criterion to predict whether an optimal search strategy exists at all, possibly also identifying the optimal balance between local and non-local moves required to place oneself in such regime. Unfortunately, the exact dependence of 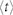 on 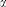 is hidden in the inverse of the matrix 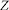 which, in general, cannot be computed explicitly. However, a principle of this sort can be formulated by resorting to a perturbative approach. If we assume that the stationary point is located at sufficiently small values of 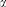, then we may consider a search time of the form
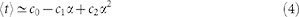
In this case, the coefficients 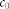, 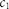 and 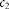, which depend on the topology of the network, can be computed analytically (see Methods). A necessary and sufficient condition for a meaningful minimum to occur is 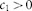 and 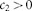 with 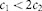, which ensures that 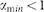. This provides a handy rule to enquire about the existence of an optimality condition for any given network.

**Figure 3:**
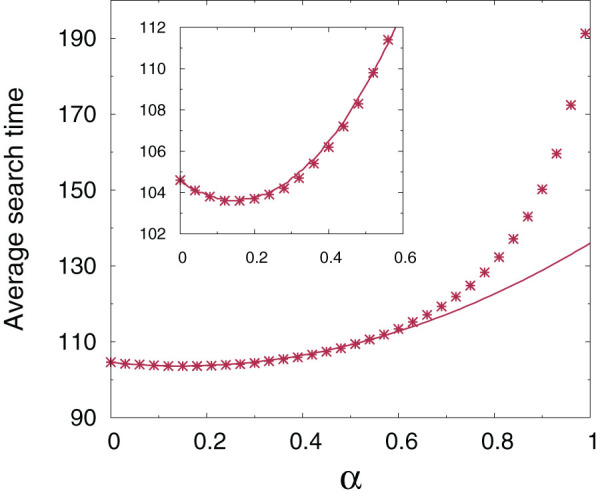
Formula (4) provides a convenient tool to enquire about the existence of an optimality criterion for the search time in a given network. The average search time in a small random network of 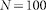 nodes (symbols) computed from Eq. (3) is compared to the approximated quadratic profile described by Eq. (4) (solid line). The inset shows a close-up of the region around the minimum. Other parameters are: 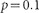, 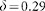.

**Figure 4:**
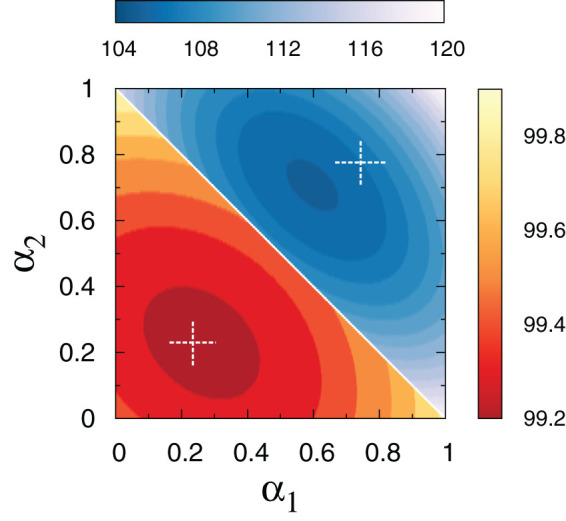
Existence of an optimum for the average search time in a multi-linked network with 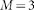. Density plot of the average search time for networks with 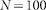 nodes and 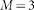. Data are obtained from formula (3), via numerical inversion of matrix (5), for every choice of 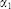 and 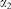. In both cases here depicted, the three matrices are generated according to the Watts-Strogatz algorithm, for different choices of the probability 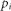 and the initial connectivity 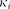, (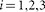): 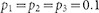, 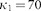, 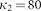, 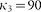 (lower triangle), 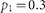, 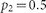, 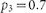, 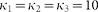 (upper triangle). The dashed crosses identify the optimal values for 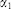 and 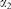 as predicted by the approximated numerical expression (8).

In all the cases that we examined, the coefficients 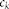 turn out to be positive. Therefore a minimum is always predicted to exist under the quadratic approximation, and closed expressions for both 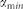 and 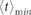 can be readily obtained. These match well the exact data computed through Eq. (3). The agreement is of course better when the minimum is found close to 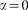 (Fig. 2–4). Explicit analytical expressions can be obtained in some limiting cases. When 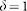 one recovers the Google matrix and the transition rate from node 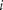 to node 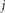 reads 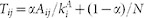. In this case it is not difficult to show that 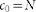, 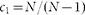 and 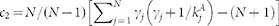, where 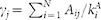 (see Supplementary Information for the full derivation). In the case of a regular lattice of connectivity 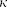, one immediately finds 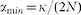 and 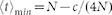. The more links per node are added, the larger the value of 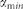 (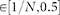), and the deeper the minimum for 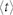 vs 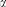. Although 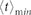 is shorter than the search time 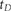 obtained for 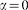, the associated correction is just 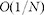.

As remarked at the beginning, the analysis carried out above can be interpreted as diffusion on graphs characterized by a single set of nodes and two independent sets of links, represented by two different adjacency matrices 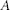 and 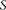. This kind of network could be termed *double-linked*. Building on this observation, we discuss in the following the generalization of our approach to arbitrary *multi-linked* networks, where an arbitrary number of independent link sets is imposed upon the same ensemble of nodes.

### The general case of *M* networks

Let us consider agents hopping on a given set of nodes, that are joined together through *M* independent sets of links, represented by as many different adjacency matrices. We term such a structure a *multi-linked* network, *i.e.* a graph where links attached to a given node have an *identity*. For example, in a transportation network two nodes (cities) could be linked through a plane connection (air link-set) and by train (railway link-set). With reference to this setting, we set out to derive the optimal coupling that maximizes the transport efficiency across the system. Again with reference to mobility applications, one can consider the effective design of an integrated urban transportation network^18^. Underground, cars and bus service define three different networks (three different link systems on the same set of nodes): their relative load in terms of users and a smart planning of the respective infrastructures could be studied via a systemic approach that builds on the formalism developed here.

In a multi-linked graph the probability of transition is specified by the following stochastic matrix 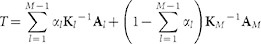 where **A***_l_* are *M* matrices of size 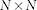, and **K***_l_* are their associated diagonal matrices of connectivities, namely 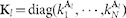. In this case the reduced matrix **Z***_j_* reads:
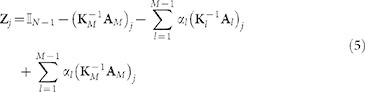
where *j* flags the position of the absorbing trap. The time it takes to reach *j* starting from 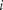 can be computed again by means of expression (2) and the average trapping time 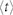 estimated through eq. (3). Following a straightforward generalization of the calculation carried out in the preceding paragraph, one can derive an approximate expression for 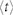 (see Methods)
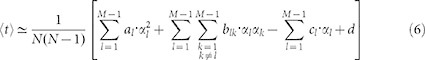
To compute the stationary point of 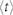, one needs to impose the condition 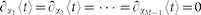, which is equivalent to setting
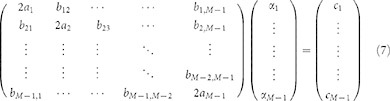
The stationary point of (6) is a global minimum if the associated Hessian matrix is positive defined.

To clarify the interest of the proposed generalization, we fix 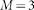 and investigate the average trapping time as a function of the two independent parameters 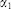 and 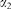. In Fig. 4 we show a density map in the 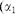, 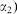 plane of the exact search time 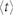 computed for two different multi-linked networks following the prescription (3). A minimum is clearly found in both cases, signaling the existence of an optimal choice of the coupling strengths that maximizes the transport efficiency. In the context of the above example, this knowledge could be exploited for the design of efficiently integrated facilities in urban transportation planning, or to favour a smart distribution of users through existing infrastructures.

Approximate analytical expressions for the optimal values of 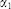 and 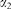 can be readily obtained from system (7), which gives
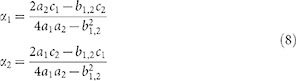
The white crosses in Fig. 4 refer to the above solution. As it happens in the case of 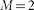 analyzed in the previous section, the approximate solution is a better estimate of the true minimum when this is found close to 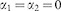.

## Discussion

Summarising, in this paper we have addressed the problem of search on networks. To this end, we have preliminarily studied the trapping problem for a modified random walk that combines local hops along the links of the graph with non-local relocation jumps, *i.e.* not along the links contained in the adjacency matrix. We have shown, both for artificial and real datasets, that an optimal balance between local and non-local moves exists which minimizes the average time required to reach a trap. Furthermore, we have derived closed analytical expressions, that enable one to predict the optimal combination as a function of the network topology.

In the second part of the paper we have focused on a random walker diffusing on a arbitrary number of interconnected networks, which share the same set of nodes but are characterized by different adjacency matrices. We have termed a complex structure of this kind a *multi-linked* network. Also in this generalized setting an optimum for the average transport time is found, which can be controlled by tuning the different coupling strengths among the individual sub-networks. In conclusion, the optimality criterion seems to be a universal dynamical mechanism, which might have exerted a critical pressure in the evolutionary selection of many naturally occurring network architectures of the multi-linked kind and that might equally well be exploited in the optimization of man-made technological solutions.

## Methods

### Computation of first passage times

The mean first passage time 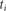, namely the time it takes for a walker starting at site 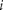 to get to any one of 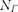 randomly placed traps, can be computed by extending to the case of a network the standard argument used in the continuum limit for a random walk on a line. Let us consider the interval 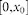 on the real axis and a random walk with two absorbing boundaries located at 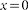 and 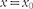. The time interval between two jumps is 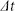 and the lattice spacing is 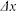. The exit time 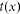 obeys to 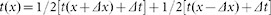 meaning that the walker can be regarded as starting one step in the future with equal probability from either 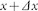 or 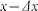. The generalization of this equation for a random walk on a network is simply given by 
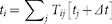
, a formulation which proves particularly convenient to investigate the trapping problem. Indeed, re-labelling the nodes of the network so as to have non-trap nodes going from 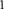 to 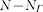 and all traps located at nodes 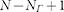 to 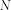, one obtains a matrix 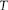 with the last 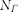 rows equal to zero. Rearranging correspondingly the array 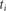 and recalling that 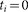 for 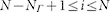, one finds that the exit times are solution of the linear system 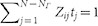, where we have denoted by 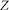 the upper-left 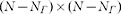 block square sub-matrix of 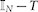. Eq. (2) is the formal solution of this last equation.

### Adjacency matrices of sub-networks from the web

To gather real data from the Web we have used the Web crawler *surfer.m* (http://www.mathworks.com/). Starting from a selected URL, the crawler identifies all the hyperlinks in the page and adds them to the list of URLs to visit. Once all these URLs are visited, the procedure is repeated recursively for each URL in the list until the assigned number of websites is reached. The outcome of the algorithm is stored in an adjacency matrix where nodes represent the visited pages: the entries of the matrix are 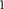 if two pages are connected trough a hyperlink, 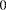 otherwise. The matrix is then symmetrized.

### Perturbative expansion of a sum of two matrices

Let 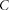 and 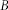 be two arbitrary non-singular square matrices of the same dimension and let us introduce the operator 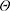, that returns the sum of all the elements of a given square matrix. Starting from the relation 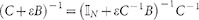, and expressing 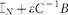 by a Neumann series^41^, it follows 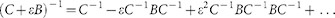. To apply this approximation to Eq. (3), we introduce two diagonal matrices associated with 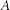 and 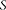, namely 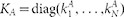 and 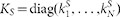. In this way, 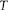 takes the form 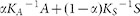. Consequently, by denoting again by 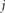 the position of the trap, the terms of the reduced matrix 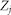 can be easily rearranged by collecting together those proportional to 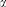. In formulae: 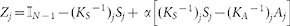. Setting 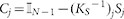, 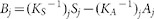 and 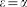, and applying the operator 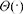 to 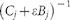, we recover Eq. (4) with 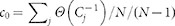, 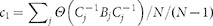 and 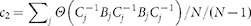.

### Generalization to the case of 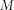 matrices

Following the same perturbative scheme detailed above, one can expand the inverse of 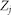 defined in eq. (5) as: 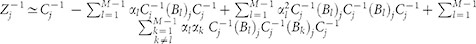.

Applying the operator 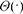 and summing over all trap locations, one obtains expression (6) for 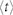, where: 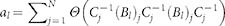, 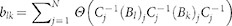, 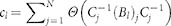 and 
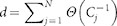
.

## Author Contributions

F. D. P., D.F. and F. P designed the study and carried out the analysis. F. D. P. carried out the numerical calculations. All authors contributed to the writing of the manuscript.

**Supplementary Information** accompanies this paper at http://www.nature.com/scientificreports

## Supplementary Material

Supplementary Information Supplementary information
