# Supplementary material for: Optimal search strategies on complex multi-linked networks
Source: Sci Rep. 2015 May 7;5:9869. doi: 10.1038/srep09869 (PMC4423499; doi:10.1038/srep09869)
Supplement: Supplementary Information [file srep09869-s1.pdf]

Optimal search strategies on complex multi-linked networks: Supplementary information

Francesca Di Patti[1,2], Duccio Fanelli[1,2], and Francesco Piazza[3]

[1]Università degli Studi di Firenze, Dipartimento di Fisica e Astronomia and CSDC, via G. Sansone 1, 50019 Sesto Fiorentino, Firenze, Italia , INFN, Sezione di Firenze, Italia
[2]INFN, Sezione di Firenze, Italia
[3]Université d'Orléans, Centre de Biophysique Moléculaire, CNRS-UPR4301, Rue C. Sadron, 45071, Orléans, France

The scaling of $\langle t \rangle$ versus N and δ

With reference to a Watts-Strogatz network with $p = 0.5$ (see lower panel of Fig. 1 in the paper), we monitored $\langle t \rangle$ versus N for different choices of the relocation density δ. The results are displayed in Fig. 1. As one can appreciate by visual inspection, α_{min} decreases as N is increased , while keeping δ fixed. At variance, working at constant N, it is possible to change the value of $\alpha_{\min}$ by tuning δ, as it can be also deduced from Fig. 1 in the main body of the paper. Interestingly, the density of long-range links δ should be reduced, to keep the optimum α unchanged, when the N gets larger. Remarkably, the gain of the assisted search over standard diffusion is preserved, if N and δ are simultaneously adjusted so as to keep $\alpha_{\min}$ fixed. Similar conclusions can be reached when operating with the scale free network.

Trapping time for the Google Matrix

According to the methods described in the main text, the analytical trapping time $\langle t \rangle$ can be approximated through

$$\langle t \rangle \simeq \frac{1}{N(N-1)} \sum_{j=1}^{N} \left[\Theta \left(\mathbf{C}_j^{-1} \right) - \Theta \left(\mathbf{C}_j^{-1} \mathbf{B}_j \mathbf{C}_j^{-1} \right) \alpha + \Theta \left(\mathbf{C}_j^{-1} \mathbf{B}_j \mathbf{C}_j^{-1} \mathbf{B}_j \mathbf{C}_j^{-1} \right) \alpha^2 \right] \tag{1}$$

with $\mathbf{C}_j = \mathbf{I}_{N-1} - (\mathbf{K}_\mathbf{S}^{-1})_j \mathbf{S}_j$ and $B = (\mathbf{K}_\mathbf{S}^{-1})_j \mathbf{S}_j - (\mathbf{K}_\mathbf{A}^{-1})_j \mathbf{A}_j$, where the subscript j indicates that the j-th row and j-th column of the matrix have been suppressed. For the Google matrix case, $\mathbf{S}$ is a full matrix where each element is equal to 1 and, consequently, $\mathbf{C}_j = \mathbf{I}_{N-1} - \mathbf{S}_j/N$. The inverse of $\mathbf{C}_j$ can be easily computed and reads $\mathbf{I}_{N-1} + \mathbf{S}_j$.

Let us now analyse the three terms of Eq. (1). To this end, from now on, we will assume $\mathbf{A}$ to be symmetric, with entries 0 or 1, and with $A_{ii} = 0$ $\forall i = 1, \dots, N$.

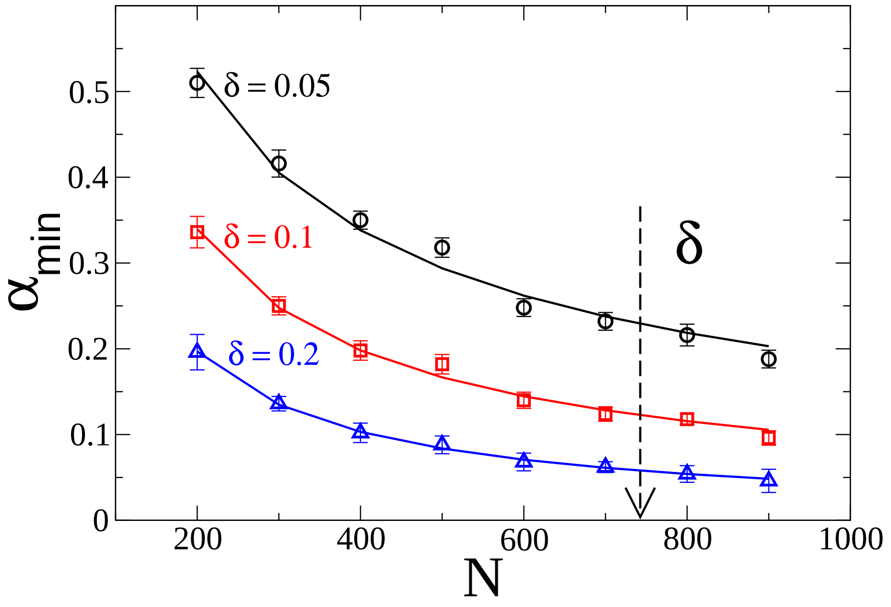

Figure 1: The average search time $\langle t \rangle$ versus N for a Watts-Strogatz network with $p = 0.5$. Different curves refer to different choices of δ, as detailed in the legend. The solid lines represent power law fits of the type a/N^b, with a and b adjustable parameters. The best fit values for b range in the interval $[0.6, 0.9]$ for the inspected values of δ.

For the first term we have

$$\Theta\left(\mathbf{C}_j^{-1}\right) = \Theta\left(\mathbf{I}_{N-1} + \mathbf{S}_j\right) = N - 1 + (N-1)^2$$

and thus

$$\frac{1}{N(N-1)} \sum_{j=1}^{N} \Theta\left(\mathbf{C}_j^{-1}\right) = \frac{1}{N(N-1)} \sum_{j=1}^{N} \left[N - 1 + (N-1)^2\right] = N \quad . \tag{2}$$

To simplify the notation, we set $\mathbf{W}_j = (\mathbf{K}_{\mathbf{A}}^{-1})_j \mathbf{A}_j$. Observing that $\mathbf{S}_j^2 = (N-1)\mathbf{S}_j$, $\mathbf{S}_j^3 = (N-1)^2 \mathbf{S}_j$ and $\Theta(a\mathbf{S}_j) = a(N-1)^2$ for $a \in \mathrm{R}$, the second

term of Eq. (1) becomes

$$
\begin{aligned}
& \frac{1}{N(N-1)}\sum_{j=1}^{N}\Theta\left(\mathbf{C}_j^{-1}\mathbf{B}_j\mathbf{C}_j^{-1}\right) \\
= & \frac{1}{N(N-1)}\sum_{j=1}^{N}\left[\Theta\left(\frac{1}{N}\mathbf{C}_j^{-1}\mathbf{S}_j\mathbf{C}_j^{-1}\right)-\Theta\left(\mathbf{C}_j^{-1}\mathbf{W}_j\mathbf{C}_j^{-1}\right)\right] \\
= & \frac{1}{N(N-1)}\sum_{j=1}^{N}\left[\Theta\left(N^2\mathbf{S}_j\right)-\Theta\left(\mathbf{C}_j^{-1}\mathbf{W}_j\mathbf{C}_j^{-1}\right)\right] \\
= & \frac{1}{N(N-1)}\left[N^2(N-1)^2-\sum_{j=1}^{N}\Theta\left(\mathbf{C}_j^{-1}\mathbf{W}_j\mathbf{C}_j^{-1}\right)\right] \\
= & N(N-1)-\frac{1}{N(N-1)}\sum_{j=1}^{N}\Theta\left(\mathbf{C}_j^{-1}\mathbf{W}_j\mathbf{C}_j^{-1}\right) \\
= & N(N-1)-\frac{1}{N(N-1)}\sum_{j=1}^{N}\sum_{\substack{i,l=1\\ i,l\neq j}}^{N}\left[N+\sum_{\substack{m=1\\ m\neq j}}^{N}\frac{A_{ml}}{k_m^A}-\sum_{\substack{m=1\\ m\neq j}}^{N}\frac{A_{mj}}{k_m^A}+\frac{A_{il}}{k_i^A}-\frac{A_{ij}}{k_i^A}\right] \\
= & N(N-1)-\frac{1}{N(N-1)}\sum_{j=1}^{N}\left[N^2(N-1)-N^2\gamma_j\right] \\
= & \frac{N}{N-1} \qquad (3)
\end{aligned}
$$

where $\gamma_j = \sum_{i=1}^{N} A_{ij}/k_i^A$, and where in the fifth row we made use of the explicit form of the element of matrix $\mathbf{C}_j^{-1}\mathbf{W}_j\mathbf{C}_j^{-1}$.

To calculate the third term of Eq. (1), it is convenient to re-write $\mathbf{C}_j^{-1}\mathbf{B}_j\mathbf{C}_j^{-1}\mathbf{B}_j\mathbf{C}_j^{-1}$ as follows

$$
\begin{aligned}
\mathbf{C}_j^{-1}\mathbf{B}_j\mathbf{C}_j^{-1}\mathbf{B}_j\mathbf{C}_j^{-1} = \; & N(N-1)\mathbf{S}_j - N\mathbf{S}_j\mathbf{W}_j - 2N\mathbf{S}_j\mathbf{W}_j\mathbf{S}_j - N\mathbf{W}_j\mathbf{S}_j \\
& +\mathbf{W}_j^2 + \mathbf{W}_j^2\mathbf{S}_j + \mathbf{S}_j\mathbf{W}_j^2 + \mathbf{S}_j\mathbf{W}_j^2\mathbf{S}_j + \mathbf{W}_j\mathbf{S}_j\mathbf{W}_j \\
& + \left(\mathbf{W}_j\mathbf{S}_j\right)^2 + \left(\mathbf{S}_j\mathbf{W}_j\right)^2 + \left(\mathbf{S}_j\mathbf{W}_j\right)^2\mathbf{S}_j \quad .
\end{aligned}
$$

We then apply the operator Θ to each term in the previous expression. Since $\Theta(\mathbf{S}_j\mathbf{W}_j) = \Theta(\mathbf{W}_j\mathbf{S}_j) = (N-1)\Theta(\mathbf{W}_j)$ and $\Theta(\mathbf{S}_j\mathbf{W}_j\mathbf{S}_j) = (N-1)^2\Theta(\mathbf{W}_j)$, we get

$$
\begin{aligned}
\Theta\left(\mathbf{C}_j^{-1}\mathbf{B}_j\mathbf{C}_j^{-1}\mathbf{B}_j\mathbf{C}_j^{-1}\right) = \; & N(N-1)^3 - 2N^2(N-1)\Theta(\mathbf{W}_j) \\
& + N^2\Theta(\mathbf{W}_j^2) + N^2\Theta(\mathbf{W}_j\mathbf{S}_j\mathbf{W}_j) \quad . \qquad (4)
\end{aligned}
$$

Let us now focus on $\Theta(\mathbf{W}_j)$, $\Theta(\mathbf{W}_j^2)$ and $\Theta(\mathbf{W}_j\mathbf{S}_j\mathbf{W}_j)$. The calculation of $\Theta(\mathbf{W}_j)$ is simple and results in

$$
\Theta(\mathbf{W}_j) = N - 1 - \gamma_j \quad . \qquad (5)
$$

To provide a closed expression for $\Theta(\mathbf{W}_j^2)$, we have first to observe that a generic element (i, l) (with $i \neq j$ and $l \neq j$) of the matrix $\mathbf{W}_j^2$ is given by

$\sum_{m\neq j} A_{im}A_{ml}/k_i^A/k_m^A$. It follows that

$$
\begin{aligned}
\Theta(\mathbf{W}_j^2) &= \sum_{\substack{i,l=1\\ i,l\neq j}}^{N} \sum_{\substack{m=1\\ m\neq j}}^{N} \frac{A_{im}}{k_i^A}\frac{A_{ml}}{k_m^A} \\
&= N-1-\gamma_j-\sum_{m=1}^{N}\frac{A_{mj}}{k_m^A}\gamma_m+\frac{\gamma_j}{k_j^A} \quad . \qquad (6)
\end{aligned}
$$

In the same way, the generic element of matrix $\mathbf{W}_j\mathbf{S}_j\mathbf{W}_j$ reads $(1-A_{ij}/k_i^A)\sum_{m\neq j} A_{ml}/k_m^A$ and thus,

$$
\begin{aligned}
\Theta(\mathbf{W}_j\mathbf{S}_j\mathbf{W}_j) &= \sum_{\substack{i,l=1\\ i,l\neq j}}^{N}\left(1-\frac{A_{ij}}{k_i^A}\right)\sum_{\substack{m=1\\ m\neq j}}^{N}\frac{A_{ml}}{k_m^A} \\
&= (N-1-\gamma_j)^2 \quad . \qquad (7)
\end{aligned}
$$

Substituting Eqs. (5)-(7) into Eq. (4) and summing over j, we find

$$
\frac{1}{N(N-1)}\sum_{j=1}^{N}\Theta\left(\mathbf{C}_j^{-1}\mathbf{B}_j\mathbf{C}_j^{-1}\mathbf{B}_j\mathbf{C}_j^{-1}\right)=\frac{N+1}{N-1}\left[-N+\sum_{j=1}^{N}\gamma_j\left(\gamma_j+\frac{1}{k_j^A}\right)\right] \quad . \qquad (8)
$$

Finally, by inserting Eqs. (2), Eq. (3) and Eq. (8) into Eq. (1) we get the following explicit expression of $\langle t\rangle$

$$
\langle t\rangle \simeq N-\frac{N}{N-1}\alpha+\frac{N+1}{N-1}\left[-N+\sum_{j=1}^{N}\gamma_j\left(\gamma_j+\frac{1}{k_j^A}\right)\right]\alpha^2 \quad .
$$
